# Actuarial Analysis of Survival after Breast Cancer Diagnosis among Lithuanian Females

**DOI:** 10.3390/healthcare12070746

**Published:** 2024-03-29

**Authors:** Justina Levickytė, Aldona Skučaitė, Jonas Šiaulys, Rokas Puišys, Ieva Vincerževskienė

**Affiliations:** 1 Institute of Mathematics, Vilnius University, Naugarduko 24, LT-03225 Vilnius, Lithuania; justina.levickyte@mif.stud.vu.lt (J.L.); aldona.skucaite@mif.vu.lt (A.S.); rokas.puisys@mif.stud.vu.lt (R.P.); 2 Laboratory of Clinical Oncology, National Cancer Institute, Santariškių 1, LT-08660 Vilnius, Lithuania; ieva.vincerzevskiene@nvi.lt

**Keywords:** breast cancer, central death rate, exposure to risk, Kaplan–Meier estimate, survival analysis, stratified Cox model, cancer awareness campaign

## Abstract

Breast cancer is the most common cause of mortality due to cancer for women both in Lithuania and worldwide. The chances of survival after diagnosis differ significantly depending on the stage of disease at the time of diagnosis and other factors. One way to estimate survival is to construct a Kaplan–Meier estimate for each factor value separately. However, in cases when it is impossible to observe a large number of patients (for example, in the case of countries with lower numbers of inhabitants), dividing the data into subsets, say, by stage at diagnosis, may lead to results where some subsets contain too few data, thus causing the results of a Kaplan–Meier (or any other) method to become statistically incredible. The problem may become even more acute if researchers want to use more risk factors, such as stage at diagnosis, sex, place of living, treatment method, etc. Alternatively, Cox models can be used to analyse survival data with covariates, and they do not require the data to be divided into subsets according to chosen risks factors (hazards). We estimate the chances of survival for up to 5 years after a breast cancer diagnosis for Lithuanian females during the period of 1995–2016. Firstly, we construct Kaplan-Meier estimates for each stage separately; then, we apply a (stratified) Cox model using stage, circumstance of diagnosis, and year of diagnosis as (potential) hazards. Some directions of further research are provided in the last section of the paper.

## 1. Introduction

Breast cancer is one of the most common forms of cancer for women despite the fact that incidence and mortality rates may differ significantly in different countries. For example, in the USA, breast cancer accounts for 30% of all new female cancers each year (see [1]), while worldwide, breast cancer is the most commonly diagnosed cancer type, accounting for 1 in 8 new cancer diagnoses (see [2]). More statistics about the morbidity and mortality of breast cancer worldwide can be found in [3] and in the references therein.

Despite advances in medicine and preventive diagnostics, which allow cancer to be detected at earlier stages and treated with modern therapies, cancer deaths remain the second leading cause of mortality among women in Lithuania. In 2022, deaths due to cancer accounted for 16.1% of all deaths among females of all ages, while in 2018, the share of deaths due to cancer was 18%. Even in 2020, when the COVID-19 pandemic began, 16.7% of all females died due to cancer, and “only” 4.8% of female deaths were due to COVID-19. Only during 2021 did cancer become the third leading cause of death among females (14%), while COVID-19 temporarily became the second leading cause (15%). For more statistics, interested readers are referred to [4].

It is not surprising that a significant amount of research has been conducted to estimate survival after a cancer diagnosis. Survival depends very much on the stage at diagnosis, as well as other factors (comorbidities, treatment received, etc.). Kaplan–Meier (KM) estimates remain one of the most popular tools for the analysis of survival after diagnosis because the Kaplan–Meier method is relatively simple to use and to obtain estimates of survival functions with; see [5,6,7,8,9,10,11,12,13], for instance.

However, one disadvantage of the KM method is that if researchers are interested in how mortality (survival) depends on various hazards (risk factors), then all observations need to be divided into subgroups (strata), and the Kaplan–Meier procedure must then be applied to each subset separately.

To avoid the problem of dividing data into subsets, researchers may use the Cox proportional hazards method. This method is widely used to model survival after diagnosis depending on various risk factors (hazards). For instance, in [14,15,16,17], the Cox proportionate hazards method was used for a survival analysis. An interesting approach was given by Putter et al. [18], who proposed a method to deal with time-dependent variables when using the Cox model for survival analysis among lung cancer patients.

In this paper, we identify the main hazards (factors) that may influence the odds of survival after breast cancer is diagnosed. We first obtain Kaplan–Meier estimates and then use the stratified Cox model to determine the impact of some potential hazards, such as stage at diagnosis, circumstance of diagnosis, and calendar year of diagnosis, on survival probabilities.

The rest of our paper is organized as follows. In Section 2, we provide a short summary of the literature and state the main goal of this study. In Section 3, we present some mathematical preliminaries and notations, which we use later, as well as the mathematical basis of the Kaplan–Meier and stratified Cox models. In Section 4, we describe the data used in our analysis. The main results of our analysis are presented in Section 5. The possible applications of our research are discussed in the concluding Section 6.

## 2. Aim and Reasearch Overview

Recently, many studies have been conducted on the survival of patients with breast cancer. These statistical survival studies are conducted in order to determine the danger of this critical disease, compare treatment methods, and determine the effectiveness of health care in a given country; see [19,20,21,22,23], for instance.

However, to our knowledge, there is little research on the survival of breast cancer patients diagnosed in Lithuania. Most studies investigate survival in cases where specific treatments were applied and/or specific genes were (or were not) observed; see [24,25], for instance. Ivanauskienė et al. [26] investigated the survival of women with breast cancer in the Kaunas region of Lithuania and estimated the probability they would survive for 1 and 2 years after diagnosis depending on the stage of their cancer and their age. None of these above-mentioned studies deal with nationwide statistics. Some statistics on incidence and survival in Lithuania may be found in [27], where a comparison of incidence and survival across European countries is given.

Interesting results may be found in [28], where data from Latvia, a country that neighbours Lithuana with a similar population and history, are given. The authors of this study estimate the three-year and five-year survival rates of patients diagnosed with breast cancer at Riga’s Pauls Stradiņš Clinical University Hospital and compare their results with nationwide data from Latvia, as well as the USA and UK.

Skucaite et al. [29] analysed survival after breast cancer diagnosis among Lithuanian females. The authors of this paper applied the Kaplan–Meier procedure to obtain survival rates and then fit appropriate analytic functions to the obtained estimates. This methodology allowed the researchers to obtain estimations of survival over longer periods. However, this research was limited to the KM method only, and the impact of specific hazards on survival was not estimated. In this paper, we go further in this direction and apply not only the KM procedure but also the (stratified) Cox model to obtain more accurate results.

The main goal of this paper is twofold. First, we aim to estimate the five-year survival rate of breast cancer patients in Lithuania (nationwide) depending on the following hazards: disease stage, circumstance of diagnosis, and period of diagnosis. Second, we also analyse the impact of these hazards on survival probability. We analyse quite recent data, i.e., cases diagnosed during the period of 1995–2016 and deaths (survivals) until the end of 2021.

## 3. Some Notations and Mathematical Preliminaries

Consider a person who has just been diagnosed with cancer. Her future lifetime is a nonnegative random variable, which we define by *T*. We assume that the survival function of lifetime *T*

S(t)=P(T>t)

is absolutely continuous.

As usual, we denote by _*h*_$p_{t}$ the probability to survive until time t+h for an individual alive at time *t* by

$${\;}_{h}p_{t}=P\left(T>t+h|T>t\right)=\frac{S(t+h)}{S(t)}.$$

Alternatively, the probability of death at time t+h after being alive at time t is defined by the equality

$${\;}_{h}q_{t}=1-{\;}_{h}p_{t}=\frac{S(t)-S(t+h)}{S(t)}.$$

The instantaneous rate of mortality, the so-called *hazard rate at time t*, or the *force of mortality at time t*, is defined by the following equality

$$\mu \left(t\right)=-\frac{S^{\prime }\left(t\right)}{S(t)},$$

which makes sense because the survival function *S* is supposed to be absolutely continuous.

### 3.1. Kaplan–Meier Estimates

Kaplan–Meier estimates are one of the most popular methods used to estimate survival probabilities. In brief, the observation interval is divided into subintervals by partitioning it at each point where a death occurs. Estimate of the survival function *S* is then obtained using the formula:

$$\hat{S}\left(t\right)=\prod_{t_{i}⩽t}\left(1-\frac{d_{t_{i}}}{l_{t_{i}^{-}}}\right),$$

where $d_{t_{i}}$ is the number of deaths that occurred at time $t_{i}$ and $l_{t_{i}^{-}}$ is the number of patients under observation alive immediately before time $t_{i}$.

For the estimate of the survival function, $\hat{S}\left(t\right)$, it is possible to calculate the approximate standard error using the Greenwood formula:

$$\sigma \left(\hat{S}\left(t\right)\right)\approx \hat{S}\left(t\right)\sqrt{\sum_{t_{i}⩽t}\frac{d_{t_{i}}}{l_{t_{i}^{-}}\left(l_{t_{i}^{-}}-d_{t_{i}}\right)}}.$$

The popularity of the Kaplan–Meier method may be explained by its (relative) simplicity and other advantages. It is suitable for datasets with a limited number of cases, such as medical trials, when traditional actuarial procedures, e.g., those used to construct a life table, may not be suitable. Despite being non-parametric, the Kaplan–Meier estimator is still a statistical estimator; hence, the standard error and confidence intervals can be calculated. Moreover, the Kaplan–Meier method allows one to estimate the death probability _*h*_$q_{t}$ when the interval *h* is as short as one day, e.g., h=1/365. Moreover, the interval *h* may differ for different subintervals and is not determined a priori by an analyst but is based on data under investigation, so *h* is determined a posteriori.

The main disadvantage of the Kaplan-Meier method is that it struggles to effectively incorporate risk factors and their impact on survival. Suppose we are interested in survival after cancer diagnosis based on stage, circumstance of diagnosis, and period of diagnosis. In such a case, all the data must be divided into 16 groups, and calculations should be repeated for each subset (see more in Section 4). This may not only become time consuming, but it may happen that some subsets will include quite a small number of observations and deaths, so a statistical analysis of such subsets will become meaningless.

More information on the Kaplan–Meier method can be found in [30,31,32] or other textbooks on statistics and/or survival theory.

We used the R software environment and its packages *Survival* and *Survminer* [33,34] to obtain Kaplan–Meier estimates. We present the R scripts used for calculations in Appendix A.

### 3.2. Cox Proportional Hazard Model

In most cases, we will observe several hazards that may affect the risk of dying. For example, when analyzing mortality in the case of a breast cancer diagnosis, the time since diagnosis may be considered the primary factor, but the stage at diagnosis, the year of diagnosis, and the patient’s age at diagnosis may also significantly influence the risk of dying. We will call such factors hazards, or *covariates*. As we already mentioned, one way is to divide all data into subsets according to the chosen covariates, but such an approach has its disadvantages. The Cox models described below offer an alternative approach to data grouping (stratification).

#### 3.2.1. Univariate and Multivariate Cox Model

An alternative method is to include covariates in the model of the hazard rate (force of mortality). A covariate may be any factor influencing survival, and covariates may be discrete or continuous variables. Typical examples of covariates may be the age of the patient, sex, stage of disease, treatment applied, etc. Suppose that in the general case we have *m* covariates. Then, for every observed individual *i*, we define the covariate vector

$$z_{i}=\left(z_{i1},z_{i2},\ldots ,z_{im}\right)$$

and the hazard rate (force of mortality) for an individual *i* becomes a function of the covariates as well as the time since diagnosis:

$$\mu_{i}\left(t\right)=\mu \left(t,z_{i}\right).$$

You may find a further explanation about the covariates used in our study in Section 5.2.

According to the Cox proportional hazard model, we suppose that the force of mortality has the following form:

(1) $$\mu \left(t,z_{i}\right)=\mu \left(t\right)e^{\beta_{1}z_{i1}+\beta_{2}z_{i2}+\ldots +\beta_{m}z_{im}},$$

where $\beta_{i},i\in \left\{1,2,\ldots ,m\right\}$ are real coefficients.

The first factor μ(t) is a function of time since diagnosis, which is called a *baseline hazard*. In usual actuarial practice, a baseline hazard is also a function of age *x*; see [35], for instance. However, in our case, it is more natural to define a baseline hazard as a function of time since diagnosis. The second factor

$$e^{\beta_{1}z_{i1}+\beta_{2}z_{i2}+\ldots +\beta_{m}z_{im}}$$

depends on covariates of the *i*-th individual, but not on time. The coefficients $\beta_{i},i\in \left\{1,2,\ldots ,m\right\}$ and the baseline hazard should be found (estimated) from data.

So, the hazard rates (hazard ratios) for two individuals, say *i* and *j*, are supposed to be in the same proportion at all times, *t*, namely

(2) $$HR\left(i,j\right)=\frac{\mu (t,z_{i})}{\mu (t,z_{j})}=\frac{e^{\beta_{1}z_{i1}+\beta_{2}z_{i2}+\ldots +\beta_{m}z_{im}}}{e^{\beta_{1}z_{j1}+\beta_{2}z_{j2}+\ldots +\beta_{m}z_{jm}}}.$$

Regression coefficients $\beta_{l},l\in \left\{1,2,\ldots ,m\right\}$ may be found by maximizing the partial likelihood function. To estimate a baseline hazard function, a researcher usually needs to make an assumption that the baseline hazard follows some known parametric law of mortality, say, Gompertz. Alternatively, if a researcher is interested in only the effects of covariates, the baseline hazard function may not be estimated since it cancels out, as may be seen from (2). The Cox model is a quite well-known example of the latter approach; see [36].

The Cox model may be applied for any single covariate l∈{1,2,…,m} (univariate Cox model) or for more than one or all covariates at the same time (multivariate Cox model).

Suppose a univariate Cox model is used for the covariate sex. Let us assume the hazard “patient is male” will be denoted by 0. Then, the hazard defined as “patient is female” will be denoted by 1. The Cox model allows us to estimate how much the risk of dying is increased (decreased) for females compared to males, but without an estimation of the baseline hazard function, it is impossible to estimate the survival function either for males or for females. In the general case, the Cox model allows one to estimate how much the risk of dying is increased (decreased) for a person with a vector of covariates $z_{i}$ compared to the risk of a person with a vector of covariates $z_{j}$. Firstly, the regression coefficients $\beta_{l},l\in \left\{1,2,\ldots ,m\right\}$ are estimated. Then, the hazard ratio (HR) for any two covariates (covariate vectors if a multivariate model is used), e.g., $z_{i}$ and $z_{j}$, are calculated. If needed, a decrease in the risk of dying may be calculated by the inverse of hazard ratio, e.g., 1/HR.

One of the main assumptions in the Cox model is that the regression coefficients β and, consequently, the hazard ratios, HR, should not be time-dependent (the hazard ratio for two covariates should remain in the same proportion during the entire observation period). So, the assumption of proportionality should be tested. This may be performed, for example, using the method of Schoenfeld residuals. The mathematics underlying this method are quite complex. The full description of this method can be found in [37], for instance. If the assumption of proportionality does not hold, the Cox model may be used; however, the results should be interpreted with great care.

The technical estimation of the regression coefficients β and the Schoenfeld residuals is much more complex and time-consuming than calculations for the Kaplan–Meier method. We used the software environment R and its packages *Survival* and *Survminer* [33,34] for calculations; see also Appendix A.

#### 3.2.2. Stratified Cox Model

If some covariates do not follow the assumption of proportionality, then a stratified Cox model may be used. Under such an approach, all data under consideration are divided into strata based on the hazards that do not meet the proportionality assumption. The main difference of this method compared to univariate or multivariate Cox models is the way the partial likelihood function is calculated. If the stratified Cox model is used, the partial likelihood function is constructed by multiplying the likelihood functions for each strata. Since a deeper knowledge of mathematics is required, we do not provide details of calculations here. Instead, interested readers are referred to [32] or other textbooks on survival analysis.

However, one should keep in mind that, contrary to uni- or multivariate Cox models, when a baseline hazard is assumed to be same for all individuals and cancels out, the baseline hazard function (see Equation (1)) can be different for different strata. Thus, differences in survival between different strata will depend not only on the hazard rates but also on the baseline hazard function. Additional assumptions may be needed for the estimation of the baseline hazard function.

## 4. Data and Their Limitations

Data collected by the Lithuanian Cancer Registry (www.nvi.lt, accessed on 17 January 2022) were used for our analysis. The Lithuanian Cancer Registry is a population-based and nationwide registry that covers the entire territory of Lithuania, and it collects information about all new cancer cases. We analysed only cases when female patients were diagnosed with breast cancer (ICD-10 code C50) for the very first time, i.e., there was no evidence that the patient was diagnosed with any type of cancer before.

For each patient, the following data were recorded:

- Exact date of cancer diagnosis.
- Circumstance of diagnosis: due to patient’s initiative, cancer awareness program, or death certificate.
- Stage at diagnosis.
- Date of last patient inspection (follow up date).
- Exact date of death, if known.

Reporting on all new cancer cases in Lithuania is mandatory. A standard form of notification must be provided to the Lithuanian Cancer Registry from all healthcare institutions. Information about the circumstances of diagnosis is provided in the notification as a list of checkboxes. The stage at the time of diagnosis is based on the assessment of clinical and/or pathological extension of the disease using the TNM classification. For our study, we used information about stage (only the first digit), as the Cancer Registry systematically verifies the conformity of TNM classification to the stage during regular data quality assessments.

We analysed cases diagnosed during the period of 1995–2016 and observed all lives from diagnosis of disease to death or until the end of the follow-up period on 31 December 2021. We found 68 cases lost to follow-up, i.e., records with no date of death, a last date of observation earlier than 31 December 2021, and a period from diagnosis to last follow-up day shorter than 60 months (5 years). Those cases were not excluded but were treated as right-censored, e.g., the survival time for such a person was considered to be at least as long as last day of their observation. The same approach was adopted when treating all survivals until the end of the study period, namely, 31 December 2021, i.e., the survival time for those cases is known to be as long as the end of the study period. It is important to note that even patients diagnosed at the end of the diagnostic period, e.g., the end of 2016, had the chance to survive at least 5 years. We disregarded the reason of death after diagnosis since we assumed that all deaths after diagnosis are due to cancer, or at least diagnosis accelerated death.

We had an initial set of 30,479 cases. After the initial inspection, we decided to exclude 102 cases (less than 1% from all records) where the date of death coincided with the date of diagnosis. Most of these cases were situations where the cause of death on the death certificate was “breast cancer”. Thus, such patients were diagnosed earlier than their death, but it was not possible to track their survival time from their moment of diagnosis until death. We also removed 1750 cases (about 6% of all records) where the stage of disease was not recorded. Finally, beginning in 2008, cancer in different breasts (left and right) were considered as two separate diseases. Thus, 520 records (about 2% of all records) were removed (cases with an earlier date of diagnosis). Therefore, our final set of data consisted of 28,107 records (N = 28,107). A more detailed distribution of cases (records) may be found in Table 1.

Deaths that occurred later than 5 years after diagnosis were disregarded since we were interested in survival up to 5 years, so all such records were treated as censored data.

We decided to analyse three main hazard factors, namely, stage at diagnosis (1 through 4); circumstances of diagnosis (cancer awareness program vs. examination on patient’s initiative), and period of diagnosis. It is obvious that stage at diagnosis may influence survival significantly, with lower survival related to higher stage. Additionally, we assumed that cancer awareness programs help diagnose the disease at earlier stages, while patients being seen at a medical office due to their own initiative may already experience some of the symptoms that indicate that the stage of the disease may be higher. Despite the fact that only about 4% of patients in our study were diagnosed via cancer awareness programs, we decided to treat this as a hazard and analyse survival based on the circumstance of diagnosis. We use the term *cancer awareness program* to define both medical check-ups due to participation in cancer awareness programs and routine regular medical check-up. We also assumed that year of diagnosis may have a positive effect on survival due to general advances in diagnosis and treatment. Therefore, we used two data intervals: the years 1995 through 2004 and the years 2005 through 2016.

Since reporting of new cancer cases is mandatory, we treated the risk of data loss due to not reporting to be quite low. We had to remove about 8% of data: 6% due to stage being unknown and 2% where patients were diagnosed with cancer in both breasts. Our database was quite big compared to other similar studies; for example, Ivanauskienė et al. analysed data from 240 patients [26], while Salimbajevs et al. analysed 377 cases [28]. We therefore assumed that a loss of 6% of our records should not have a significant impact on our results. However, patients that were diagnosed with cancer in different breasts may have different mortality compared to those diagnosed with cancer in one breast only. We did not analyse such cases separately; however, this may be one direction for further research.

## 5. Main Results

The main results of our analysis are described in this section. We start from the Kaplan–Meier estimates and then continue with the (stratified) Cox method.

### 5.1. Kaplan–Meier Estimates

We used the Kaplan–Meier method to analyse survival after diagnosis according to risk (hazard) group: stage at diagnosis (1 through 4), circumstance of diagnosis (cancer awareness program vs. patient’s initiative) and period of diagnosis (1995 through 2004 vs. 2005 through 2016).

The main results are summarized in Table 2. We note that data were right censored, i.e., the maximum period of observation was 60 months after diagnosis. Hence, the average of the future lifetime and median of the future lifetime after diagnosis were estimated under the condition that the maximum observed period of survival was 60 months.

As expected, a higher stage at diagnosis has a negative impact on survival. Patients diagnosed with Stage 1 cancer may expect that their average survival period will be more than twice longer compared to patients diagnosed with Stage 4 cancer. Other hazards did not lead to such significant differences in average survival. However, patients diagnosed due to cancer awareness programs had a bit longer average survival time compared to those receiving check-ups at their initiative. A later period of diagnosis also had a slightly positive impact on survival.

**Figure 1:**
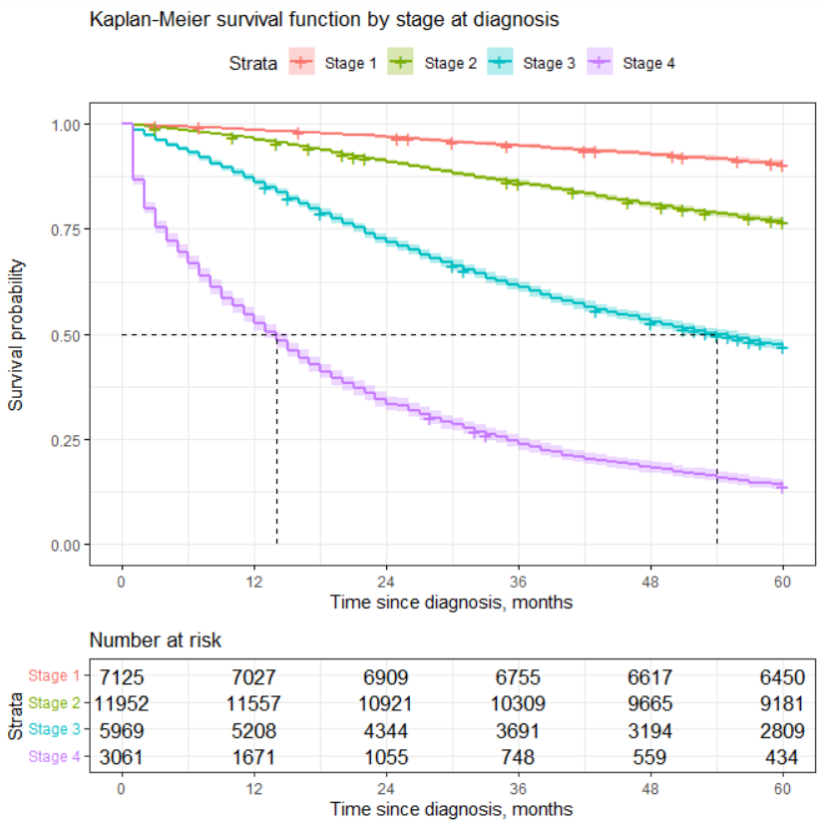
Estimation of survival by stage at diagnosis. Dash lines indicate the median survival times presented in Table 2.

We estimated the probability of surviving 5 years based on stage of disease, for instance. For patients diagnosed with Stage 1, this probability is slightly more than 90%, while the chances for those diagnosed with Stage 4 are slightly less than 14%. For more details, please see Table 3 and Figure 1.

Quite significant differences in survival according to stage may be seen from Table 3. We ran a log-rank test, which confirmed that the differences in survival according to the different stages at diagnosis are statistically significant.

As we already noted, there is not much research on the survival of breast cancer patients diagnosed in Lithuania or even in neighbouring countries. A comparison of our results to some results found by other studies are presented in Table 4.

As seen from Table 4, our results are very much in line with those obtained in [29]. We found better survival compared to data from Latvia, but this result should be interpreted with care. We analysed the most recent available data (1995–2016, with a last follow-up date of 31 December 2021), while Latvian results were from observations during 2005. As we will see, a later period of diagnosis may have quite a significant impact on survival. This same reason may be used to explain, at least partially, the difference between the overall 5-year survival found in our study compared to CONCORD-3. We used our results based on the most recent data (2010–2014) from the CONCORD-3 study, while our study is related to a much longer period with poorer survival at the beginning of the period of observation.

We then analysed survival based on circumstance of diagnosis. The main results are presented in Table 5.

**Figure 2:**
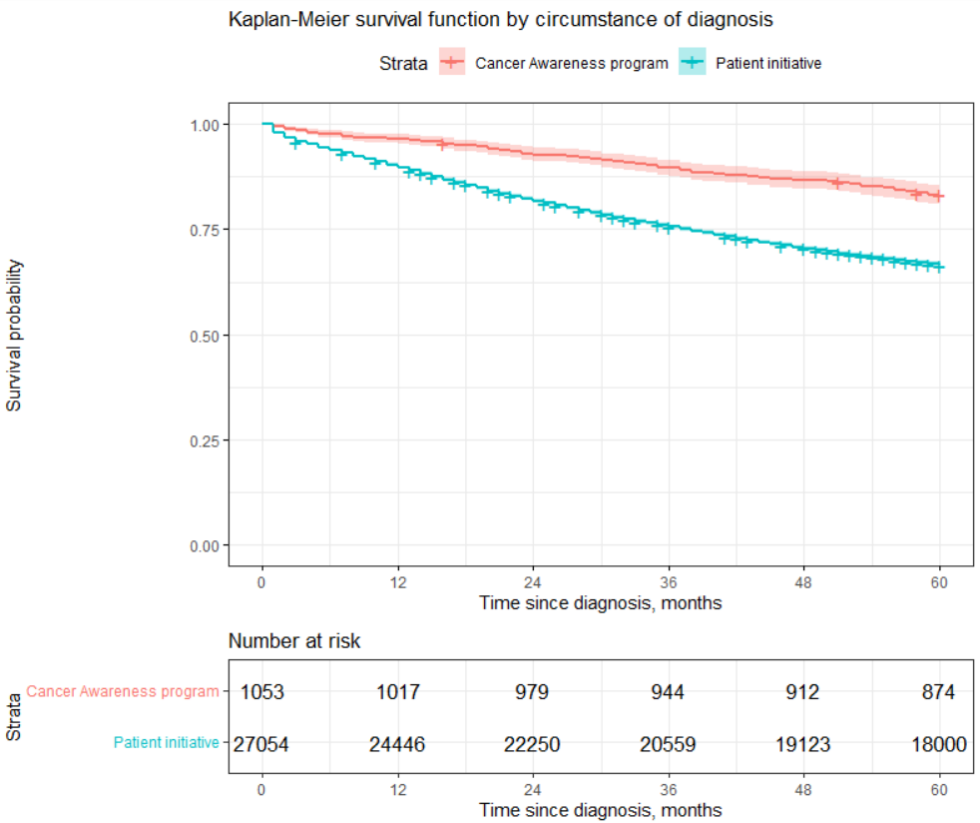
Estimation of survival by circumstance of diagnosis.

As seen from Table 5 and Figure 2, those patients who received a check-up at their initiative had a 66% chance to survive 5 years, compared to slightly more than 80% survival after a diagnosis obtained due to participation in cancer awareness programs (CAPs). The log rank test confirmed the presence of statistically significant differences in survival by circumstance of diagnosis. However, these results should be interpreted with care since only about 4% patients were diagnosed during CAPs. Moreover, it is obvious that better survival is influenced not by the method through which a diagnosis was obtained (CAPs vs. patient’s initiative) but rather by the fact that usually, those who participated in CAPs were diagnosed with lower stages of cancer, which leads to better survival rates. Readers may see from Table 6 that the percentage of patients diagnosed with Stage 1 or 2 disease due to CAPs amount to 86%, compared to 67% of cases diagnosed during an examination undertaken due to patient initiative. The most life-threatening stage of cancer, Stage 4, was diagnosed for only 3% of patients who participated in CAPs compared to 11% of patients examined due to their initiative. Our results show that it is worth investing in CAPs since they may increase the chances of being diagnosed at an earlier stage. Considering differences in life expectancy after diagnosis, the cost of treatment, and the cost of temporary disability due to disease, CAPs can not only help to prolong the lifespan of patients but also have a positive impact on public finances.

Finally, we constructed Kaplan–Meier estimates of the survival function based on the period of diagnosis. The main results are presented in Table 7.

**Figure 3:**
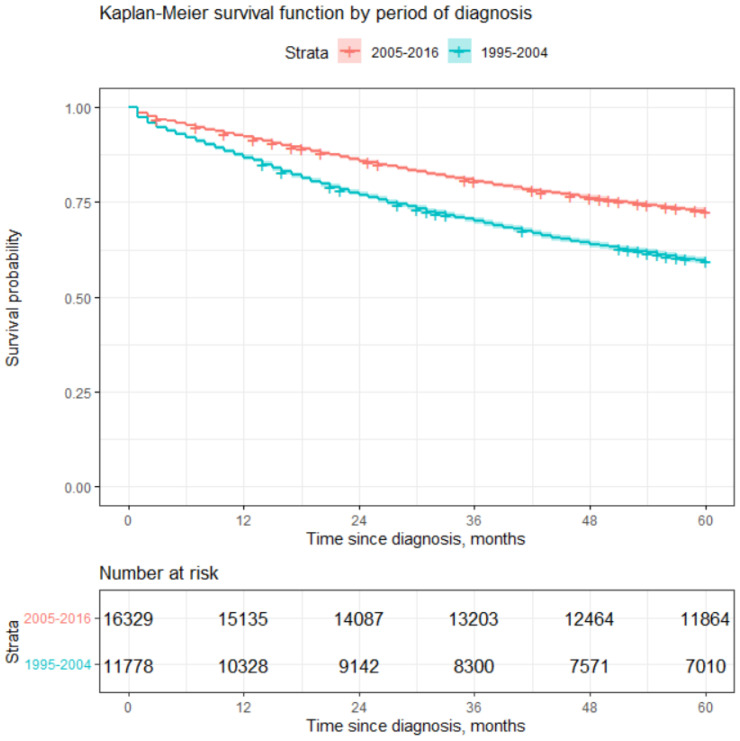
Estimation of survival by period of diagnosis.

As seen from Table 7 and Figure 3, poorer 5-year survival rates were observed for the earlier period (1995–2004). Patients diagnosed later (2005–2016) had about a 22% higher probability of surviving 5 years compared to patients diagnosed during the period of 1995–2004. A log-rank test confirmed statistically significant differences in survival. Such differences may arise due to several reasons, with the main ones being advances in medicine and the introduction of CAPs. CAPs were started in Lithuania during the period of 2002–2004 and probably led to more cases being diagnosed at earlier stages (see Table 8). Advances in medicine probably led to better survival rates after diagnosis due to new treatment methods.

### 5.2. Cox Models

We used 3 covariates in our study: stage at diagnosis, circumstance of diagnosis (examined at patient’s initiative vs. cancer awareness program) and period of diagnosis (1995–2004 vs. 2005–2016).

Since there are four possible stages of disease and because stages are measured using an ordinal scale, we used 3 dummy variables for stages 2 through 4 when defining force of mortality; see Equation (1). Each dummy variable may take a value of 1 if its corresponding stage was diagnosed or a value of 0 otherwise. We did not define dummy variable for Stage 1 cancer since Stage 1 cancer is diagnosed if all three other dummy variables take a value of 0. Stage 1 cancer is thus considered to be the baseline hazard level.

For the other two hazards, we proceeded as follows:

$$Circumstance=\left\{\begin{array}{cc} 0,\; & \mathrm{if}\;\mathrm{patient}\;\mathrm{examined}\;\mathrm{on}\;\mathrm{her}\;\mathrm{initiative};\\ 1,\; & \;\mathrm{if}\;\mathrm{patient}\;\mathrm{examined}\;\mathrm{due}\;\mathrm{CAP}. \end{array}\right.$$

$$Period=\left\{\begin{array}{cc} 0,\; & \mathrm{if}\;\mathrm{patient}\;\mathrm{diagnosed}\;\mathrm{during}\;1995–2004;\\ 1,\; & \mathrm{if}\;\mathrm{patient}\;\mathrm{diagnosed}\;\mathrm{during}\;2005–2016. \end{array}\right.$$

If the value of a particular hazard was 0, it was considered to be the baseline hazard level, and hazard ratios were calculated with respect to the baseline hazard level.

#### 5.2.1. Univariate Cox Model

We started our analysis by applying the univariate Cox model, i.e., we evaluated β and the hazard ratios for each hazard factor separately; see Table 9.

Our analysis showed that a higher stage at diagnosis significantly increases the risk of death. Compared to Stage 1 disease, the diagnosis of Stage 2 disease increases the risk of dying about 2.65 times because the hazard rate estimate is $\hat{HR}=2.65$. Comparatively, Stage 3 and Stage 4 disease increase the risk of dying by 7.62 and 23.55 times, respectively.

Patients who were diagnosed during CAPs have a lower risk of dying of 2.25=1/0.445 ($\hat{HR}=0.445$) times compared to patients examined due to their initiative. However, it is worth mentioning that usually, lower stages of cancer are found during examination via CAPs; see Table 6. This result is in line with the results presented in the previous section; see Figure 2.

A later period (2005–2016) of diagnosis decreases the risk of dying by 1.63 times ($\hat{HR}\;=\;0.612$) compared to an earlier period of diagnosis (1995–2004). This result is also in line with the result stated in the previous section; see Figure 3.

The Wald statistics showed that the impacts of all hazards are statistically significant. For more results, please refer to Table 9.

**Figure 4:**
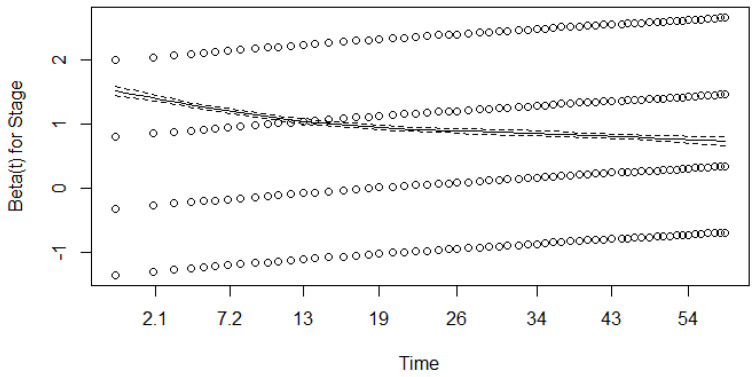
Results of the Schoenfeld test (β for the covariate of stage). Dotted lines represent results for different stages, dashed line represent results for general model.

**Figure 5:**
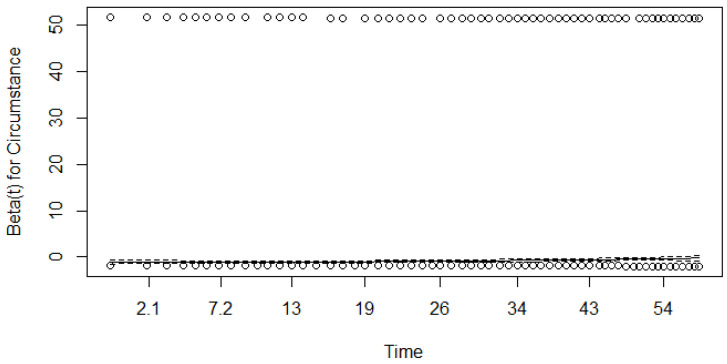
Results of the Schoenfeld test (β for the covariate of circumstance). Dotted lines represent results for different circumstances, dashed line represent results for general model.

**Figure 6:**
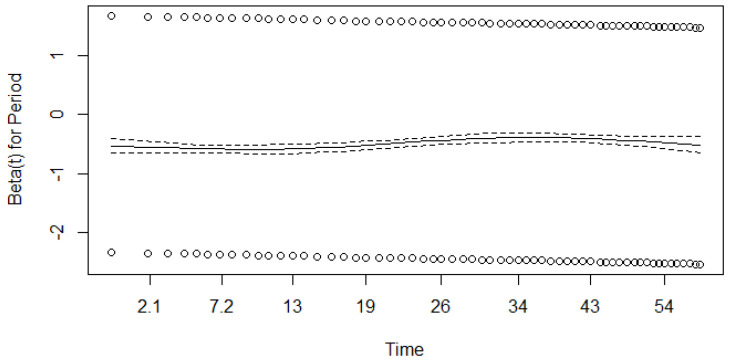
Results of the Schoenfeld test (β for a covariate of period). Dotted lines represent results for different periods, dashed line represent results for general model.

We ran a Schoenfeld test (based on residuals) to check the proportionality assumption; see Figure 4, Figure 5 and Figure 6.

As shown in Figure 4, Figure 5 and Figure 6, β for the covariate of stage is time-dependent, while the β coefficients for the other two covariates may fulfil the assumption of proportionality. To check this further, we ran a Schoenfeld test based on $\chi^{2}$ statistics and obtained *p* values; see Table 10.

As seen in Table 10, the *p*-value is less than the chosen significance level of α=0.05 for all covariates. This means that the assumption of proportionality should be rejected for all covariates (the assumption that the regression coefficients β do not depend on time should be rejected).

Since the Schoenfeld tests showed that all three covariates most likely failed to fulfil this assumption, the results of the univariate Cox model should be interpreted with care.

#### 5.2.2. Multivariate Cox Model

We constructed a multivariate Cox model using all three covariates; see Equation (1). Recall that the hazard rates of two randomly selected individuals should remain in constant proportion all the time; see Equation (2). We used the Schoenfeld test again to test the assumption of proportionality; see Table 11.

As seen from Table 11, the covariate of stage failed to fulfil this assumption of proportionality. The covariate of period fulfils this assumption, while the covariate of circumstance formally does not fulfil it. However, one may notice that for a significance level of, say, α=0.025, the covariate of circumstance will fulfil the assumption of proportionality.

Therefore, we stratified our dataset by dividing it into four strata based on stage at diagnosis and applied a stratified Cox model. Recall that the stratified Cox model allows one to perform calculations for all data at once, thus allowing us to avoid the potential issue of some strata containing too few data.

#### 5.2.3. Stratified Cox Model

The Schoenfeld test showed that the stratified Cox model fulfils the assumption of proportionality with a significance level of α=0.05; see Table 12.

As seen in Table 13, both the covariates circumstance and period of diagnosis are statistically significant. A diagnosis obtained due to a cancer awareness program and a later period of diagnosis have a positive impact on survival. Participation in CAPs may reduce the risk of dying by 1.31 times ($\hat{HR}$ = 0.763), while the later period of diagnosis reduces the risk of dying by 1.25 times ($\hat{HR}$ = 0.802).

Reduction in mortality, as determined by the stratified Cox model, is independent of stage at diagnosis (it is the same for all stages). However, recall that the baseline hazard μ(t) from Equality (1) can be different for different stages of disease. Thus, differences in survival between stages will depend not only on the hazard rates but also on the baseline hazard function. However, we did not make any assumption about the function of the baseline hazard and therefore did not estimate it.

**Figure 7:**
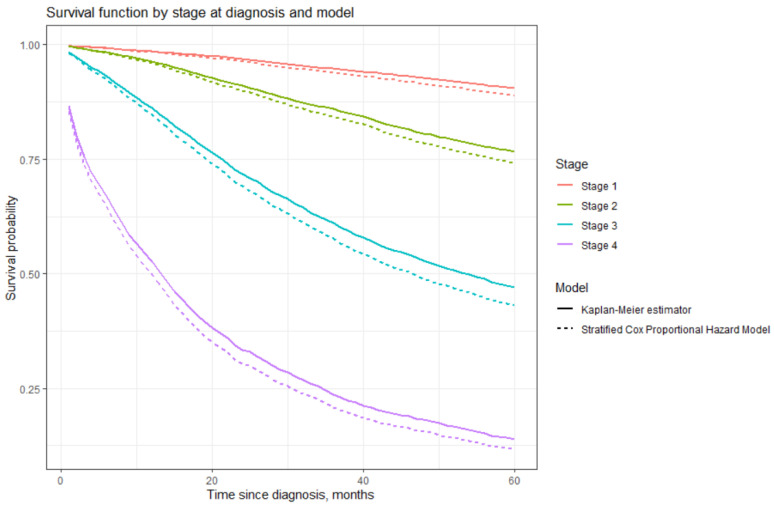
Comparison of survival functions.

We compared survival curves by stage at diagnosis obtained using the Kaplan–Meier estimator and the stratified Cox proportional hazard model; see Figure 7.

As seen in Figure 7, the results obtained using the stratified Cox model show slightly lower survival for all stages. However, survival curves for every stage (1 through 4) are parallel, depicting similar trends. Therefore, the results obtained in our study are consistent. The stratified Cox proportional hazard model reasonably and adequately reflects the relationship between covariates and survival time in our dataset.

## 6. Conclusions

We examined survival after breast cancer diagnosis among Lithuanian females. As might be expected, the stage of the disease is a very important hazard factor. Higher stages of the disease decrease life expectancy quite significantly. We examined two more hazards, circumstance of diagnosis and period of diagnosis. We found that better survival is observed if the examination of the patient was carried out during a cancer awareness program (CAP) and if the disease was diagnosed during a later period. However, CAPs increase the chances of being diagnosed at an earlier stage. On the other hand, later period of diagnosis increases the chances that the patient was examined during CAPs, as such programs were introduced in Lithuania during the period of 2002–2004. Better survival is more likely due to the earlier stage of the disease rather than participation in CAPs or the later period of diagnosis. Our results are in line with those obtained in [17], which states the following: *The main factor causing low survival time was because the patient comes for treatment already in an advanced stage even accompanied by comorbidities (such as diabetes, anemia and hypertension).*

Our analysis using a stratified Cox model showed that participation in CAPs and a later period of diagnosis have a positive impact on survival. The stratified Cox model showed slightly lower survival compared to the Kaplan–Meier method. We assume that the Cox model probably gives more accurate results since it incorporates the impact of covariates when predicting survival for different stage at diagnosis. It is worthwhile to continue research in this direction and to include more covariates, such as age at diagnosis, treatment method, comorbidities, etc.

Another suggested direction for further studies is the estimation of the baseline hazard function used in Cox models. Some ideas for such research may be found, for example, in [39]. Our analysis showed that some covariates used in the Cox model are time-dependent. In such cases, the results of the Cox model may be misleading, so methods that allow one to deal with time-dependent covariates are needed.

Finally, the survival rates for breast cancer in Lithuania are still among the worst in the world (see [38]). One reason for this may be that in Lithuania, breast cancer screening programs are still not effective; the percentage of participation in 2022 was only 26.6 percent (see [40] (in Lithuanian), [41], and references therein). It is necessary to identify the major risks that have a negative impact on survival. Making CAPs more efficient may help since the early detection of breast cancer may lead to significant positive impacts on survival rates and better quality of life after treatment.

## Figures and Tables

**Table 1:** Distribution of cases.

Stage at Diagnosis	Circumstance of Diagnosis		Period of Diagnosis	
	At Patient’s Initiative	Participation in CAP	1995–2004	2005–2016
1st	6594	531	1913	5212
2nd	11,576	376	5364	6588
3rd	5858	111	2771	3198
4th	3026	35	1730	1331
Total	27,054	1053	11,778	16,329

**Table 2:** Survival according to hazard groups.

Hazards	Cases Observed (%)	Deaths Observed (%)	Average Survival Time *	Median Survival Time **
All cases (patients)	28,107 (100%)	9249 (100%)	48.347 (1.152)	-
Stage at diagnosis				
1st	7125 (25.35%)	675 (7.30%)	57.431 (0.123)	-
2nd	11,952 (42.52%)	2788 (30.14%)	53.180 (0.134)	-
3rd	5969 (21.24%)	3155 (34.11%)	41.613 (0.271)	54 (52–57)
4th	3061 (10.89%)	2631 (28.45%)	21.468 (0.376)	14 (13–15)
Circumstance of diagnosis				
Patient’s initiative	27,054 (96.25%)	9072 (98.09%)	48.093 (0.118)	-
Cancer awareness program	1053 (3.75%)	177 (1.91%)	54.891 (0.417)	-
Period of diagnosis				
1995–2004	11,778 (41.90%)	4774 (51.62%)	45.291 (0.192)	-
2005–2016	16,329 (58.10%)	4475 (48.38%)	50.553 (0.139)	-

* Standard error is given in brackets. ** Median and its confidence interval with 95% confidence level. If the median is not given, then it means that more than half of patients survived at least 60 months.

**Table 3:** Some estimates of survival function by different stages at diagnosis.

Time Since Diagnosis in Years	Stage at Diagnosis			
	1st $\hat{S}\left(t\right)$ *	2nd $\hat{S}\left(t\right)$	3rd $\hat{S}\left(t\right)$	4th $\hat{S}\left(t\right)$
1	98.582% (0.142%)	96.318% (0.179%)	86.212% (0.518%)	52.630% (1.715%)
2	96.841% (0.214%)	91.003% (0.288%)	71.832% (0.811%)	33.420% (2.551%)
3	94.804% (0.277%)	85.935% (0.370%)	61.117% (1.033%)	23.733% (3.241%)
4	92.767% (0.324%)	80.635% (0.488%)	52.865% (0.646%)	18.153% (3.842%)
5	90.517% (0.384%)	76.654% (0.505%)	47.092% (1.373%)	13.984% (4.489%)

* Standard error is given in parenthesis.

**Table 4:** Comparison of 5-year survival based on stage of disease.

Stage at Diagnosis	Current Study	Lithuania 1995–2012 *	Latvia 2005 **	CONCORD-3 Study ***
1st	90.52%	90.22%	88%	- ****
2nd	76.65%	75.68%	74%	-
3rd	47.09%	45.43%	43%	-
4th	13.98%	13.85%	4%	-
Overall	67%	-	-	73%

* Source [29]. ** Source [28]. *** Sources [27,38]; survival rate based on observations from 2010–2014 period. **** Data are not available.

**Table 5:** Some estimates of survival function by circumstance of diagnosis.

Time Since Diagnosis	Circumstance of Diagnosis	
	On Patient’s Initiative $\hat{S}\left(t\right)$ *	CAP in Years $\hat{S}\left(t\right)$
1	89.7% (0.185%)	96.5% (0.567%)
2	81.7% (0.235%)	92.8% (0.798%)
3	75.6% (0.261%)	89.5% (0.943%)
4	70.4% (0.278%)	86.6% (1.050%)
5	66.4% (0.287%)	83.2% (1.153%)

* Standard error is given in parenthesis.

**Table 6:** Number of cases by stage at diagnosis and circumstance of diagnosis.

Stage at Diagnosis	Examined at Patients Initiative		Examined during CAPs	
	Number of Cases	Percentage of Cases	Number of Cases	Percentage of Cases
1	6594	24%	531	50%
2	11,576	43%	376	36%
3	5858	22%	111	11%
4	3026	11%	35	3%
Total	27,054	100%	1053	100%

**Table 7:** Estimates of survival function by period of diagnosis.

Time Since Diagnosis in Years	Period of Diagnosis	
	1995–2004 $\hat{S}\left(t\right)$ *	2005–2016 $\hat{S}\left(t\right)$
1	86.8% (0.311%)	92.3% (0.2092%)
2	77.0% (0.388%)	85.9% (0.2726%)
3	70.1% (0.422%)	80.5% (0.3101%)
4	63.9% (0.443%)	76.2% (0.3336%)
5	59.4% (0.453%)	72.6% (0.3493%)

* Standard error is given in parenthesis.

**Table 8:** Number of cases by stage at diagnosis and period of diagnosis.

Stage at Diagnosis	Period of Diagnosis: 1995–2004		Period of Diagnosis: 2005–2016	
	Number of Cases	Percentage of Cases	Number of Cases	Percentage of Cases
1	1913	16%	5212	32%
2	5364	46%	6588	40%
3	2771	24%	3198	20%
4	1730	15%	1331	8%
Total	11,778	100%	16,329	100%

**Table 9:** Results obtained by applying the univariate Cox proportionate hazards model.

Covariate:	Regression Coefficient $\hat{\beta }$ *	Hazard Rate $\hat{\mathrm{HR}}$ **	*p* ***
Stage:			
1st		1	
2nd	0.975 (0.043)	2.651 (2.437–2.884)	<0.001
3rd	2.030 (0.043)	7.617 (7.009–8.278)	<0.001
4th	3.15914 (0.044)	23.550 (21.626–25.646)	<0.001
Circumstance			
Patients initiative		1	
CAP	−0.810 (0.076)	0.445 (0.384–0.516)	<0.001
Period of diagnosis			
1995–2004		1	
2005–2016	−0.490 (0.021)	0.612 (0.588–0.638)	<0.001

* Standard error is given in parentheses. ** Confidence interval with 95% confidence level is given in parentheses. *** *p* of the Wald test. If *p* is lower than a confidence level α=0.05, then the covariate is statistically significant.

**Table 10:** Results of the Schoenfeld test for the univariate model.

Covariate	$\chi^{2}$ Statistics	Degrees of Freedom	*p* Value
Stage	415	3	<0.001
Circumstance	8.5	1	0.0036
Period	6.5	1	0.011

**Table 11:** Results of the Schoenfeld test for the multivariate model.

	$\chi^{2}$ Statistics	Degrees of Freedom	*p* Value
Stage	414.61	3	<0.001
Circumstance	4.92	1	0.027
Period	1.49	1	0.223
Overall	417.60	5	<0.001

**Table 12:** Results of the Schoenfeld test for the stratified Cox model.

	$\chi^{2}$ Statistics	Degrees of Freedom	*p* Value
Circumstance	1.08	1	0.30
Period	1.62	1	0.20
Overall	2.89	2	0.24

**Table 13:** Results of the stratified Cox model.

Covariate	Regression Coefficient $\hat{\beta }$ *	Hazard Ratio $\hat{\mathrm{HR}}$ **	*p* ***
Circumstance			
Patients’ initiative		1	
CAP	−0.271 (0.076)	0.763 (0.657–0.886)	<0.001
Period of diagnosis			
1995–2004		1	
2005–2016	−0.221 (0.021)	0.802 (0.769–0.836)	<0.001

* Standard error is given in parentheses. ** Confidence intervals with 95% confidence levels are given in parentheses. *** *p*-value of the Wald test. If the *p*-value is less than the confidence level α=0.05, the covariate is statistically significant.

## Data Availability

The data collected by the Lithuanian Cancer Registry were used for our analysis. The Lithuanian Cancer Registry is a nationwide and population based cancer registry that covers the whole territory of Lithuania. Ethical approval for the analysis of the aggregated and anonymized data was not required.

## Appendix A. R Scripts Used for Calculations

#Kaplan-Meier calculations
data1 <- read_excel(“C:/Users/R/Krūties vėžys 1995-2016 m.xlsx”) #Read input data
attach(data1)
km1 <- survfit( Surv(Time, Death)^~^ 1, type=“kaplan-meier”)
summary(km1)
#Time– time since diagnosis until end of observations, in months.
#Death=1 in case of death of patient; Death=0 otherwise.
survplots <- list()
survplots[[1]]<-ggsurvplot(km1,combine=TRUE, data1, title=’Visos pacientės’,
legend.title = “All cases (patients)”, legend.labs = c(“Strata”), xlim = c(0,60),
xlab=“Time since diagnosis, months”, ylab=“Survival probability”, conf.int = TRUE,
ggtheme = theme_bw(), break.time.by = 12, surv.median.line = “hv”)
#By stage
km2 <- survfit( Surv(Time, Death)^~^ Stage, type=“kaplan-meier”)
summary(km2)
fit <- list(km1 = km1, km2=km2)
survplots[[2]]<-ggsurvplot(km2, data1,title=’Kaplan - Meier survival function by stage at diagnosis’,
legend.title = “Stage”, legend.labs = c(“1”, “2”, “3”, “4”), xlim = c(0,60),
xlab=“Time since diagnosis, months”,ylab=“Survival probability”, conf.int = TRUE, pval = TRUE,
risk.table.height = 0.4,ggtheme = theme_bw(),
break.time.by = 1, surv.median.line = “hv”)
#log-rank test
surv_diff <- survdiff(Surv(Time, Death)^~^ Stage)
surv_diff
#By circumstance
km3 <- survfit( Surv(Time, Death)^~^ Circumstance, type=“kaplan-meier”)
summary(km3)
fit <- list(km1 = km1, km3=km3)
survplots[[3]]<-ggsurvplot(fit,combine=TRUE, data1,
title=’Kaplan - Meier survival function by circumstance of diagnosis’,legend.title = “Circumstance”,
legend.labs = c(“Strata”,“Patient initiative”, “Cancer awareness program”), xlim = c(0,60),
xlab=“Time since diagnosis, months”,ylab=“Survival probability”,conf.int = TRUE,
pval = TRUE, risk.table.height = 0.4,ggtheme = theme_bw(),
break.time.by = 12, surv.median.line = “hv”)
#log-rank test
surv_diff <- survdiff(Surv(Time, Death)^~^ Circumstance)
surv_diff
#By period of diagnosis
km4 <- survfit( Surv(Time, Death)^~^ Year, type=“kaplan-meier”)
summary(km4)
fit <- list(km1 = km1, km4=km4)
survplots[[4]]<-ggsurvplot(fit, combine=TRUE,data1,
title=’Kaplan - Meier survival function by period of diagnosis’, legend.title = “Metai”,
legend.labs = c(“Strata”,“1995-2004”, “2005-2016”), xlim = c(0,60),
xlab=“Time since diagnosis, months”,ylab=“Survival probability”,conf.int = TRUE,
pval = TRUE, risk.table.height = 0.4,
ggtheme = theme_bw(), break.time.by = 12, surv.median.line = “hv”)
#log-rank test
surv_diff <- survdiff(Surv(Time, Death)^~^ Year)
surv_diff
arrange_ggsurvplots(survplots, print = TRUE,ncol = 2, nrow = 2,
risk.table.height = 0.3, title=’Kaplan-Meier estimates’)
#Cox PH model
#Univariate model
res.cox1 <- coxph(Surv(Time, Death) ^~^ Stage)
summary(res.cox1)
res.zph1 <- cox.zph(res.cox1)
res.cox2 <- coxph(Surv(Time, Death) ^~^ Circumstance)
summary(res.cox2)
res.zph2 <- cox.zph(res.cox2)
res.cox3 <- coxph(Surv(Time, Death) ^~^ Year)
summary(res.cox3)
res.zph3 <- cox.zph(res.cox3)
#Schoenfeld test
plot(res.zph1)
plot(res.zph2)
plot(res.zph3)
# Multivariate Cox model
res.cox4 <- coxph(Surv(Time, Death) ^~^ Stage+Circumstance+Year)
summary(res.cox4)
res.zph4 <- cox.zph(res.cox4)
#Stratified Cox Model
res.cox5 <- coxph(Surv(Time, Death) ^~^ Circumstance+Year+strata(Stage))
summary(res.cox5)
res.zph5 <- cox.zph(res.cox5)
